# Epidemiology of human adenovirus and molecular characterization of human adenovirus 55 in China, 2009–2012

**DOI:** 10.1111/irv.12232

**Published:** 2014-01-28

**Authors:** Qing-Bin Lu, Yi-Gang Tong, Ying Wo, Hong-Yu Wang, En-Mei Liu, Gregory C Gray, Wei Liu, Wu-Chun Cao

**Affiliations:** a School of Public Health, Peking UniversityBeijing, China; b State Key Laboratory of Pathogen and Biosecurity, Beijing Institute of Microbiology and EpidemiologyBeijing, China; c Children's Hospital, Chongqing Medical UniversityChongqing, China; d Department of Environmental and Global Health, College of Public Health and Health Professions, University of FloridaGainesville, FL, USA

**Keywords:** Acute respiratory disease, children, China, human adenovirus

## Abstract

**Background:**

Human adenovirus 55 (HAdV-55) has caused recent outbreaks of acute respiratory disease (ARD) among adults and military trainees. The active surveillance for HAdV infections was sparse in China, and current knowledge on the HAdV-type distributions and its molecular evolution is lacking.

**Objectives:**

To acquire better understanding on the prevalence and molecular evolution of HAdV-55 strains in China, for an informed strategy for disease control and prevention.

**Population/Methods:**

Nasopharyngeal aspirates were collected from hospitalized children with ARTI in Chongqing during 2009–2012. The genotype of HAdV isolates were determined by sequencing the partial hexon and fiber genes. Whole genome sequences of HAdV-55 were obtained for molecular evolution analysis.

**Results:**

About 191 (8·55%) HAdV were detected in 2234 children, including 92 (48·2%) with HAdV-7, 72 (37·7%) with HAdV-3, 6 (3·1%) with HAdV-55, 5 (2·6%) with HAdV-5, 4 (2·1%) with HAdV-1, 1 (0·5%) with HAdV-2, and 11(5·8%) with untyped HAdV. Four of these children developed pneumonia, two of whom were diagnosed with severe pneumonia and/or encephalopathy. HAdV-55 isolates clustered with HAdV-11 sequences based on the hexon gene and clustered with HAdV-14 sequences based on the fiber gene and the whole genome. The overall evolutionary rates of hexon gene, fiber gene, and whole genome of HAdV-55 were estimated at 6·2 × 10^−5^ s/s/y, 8·0 × 10^−5^ s/s/y, and 1·7 × 10^−5^ s/s/y, respectively.

**Conclusions:**

This study suggested HAdV-55 as an emerging infectious disease pathogen has conserved genetic structure and is closely related to each other. Further molecular investigation based on HAdV-55 of wider origin might facilitate understanding its diversity, dissemination, and transmission in China.

## Background

Human adenoviruses (HAdV) are a common cause of acute respiratory diseases, causing sporadic infections, as well as community and institutional outbreaks.^1^ Infection with HAdV rarely causes serious or fatal illness in otherwise healthy individuals, but may cause severe disease in newborn, elderly, or immunocompromised persons.^1^ There are at least 69 recognized HAdV genotypes (http://hadvwg.gmu.edu/), which are assigned to seven subgroups (A–G) according to biophysical, biochemical, and genetic characteristics.^2^ The spectrum of clinical disease associated with HAdV is broad and depends largely upon the infecting HAdV genotype. Clinical signs and symptoms include fever, rhinorrhea, pharyngitis, conjunctivitis, gastroenteritis, bronchitis, pneumonia, acute hemorrhagic cystitis, meningoencephalitis, and rarely life-threatening disseminated diseases.^1^ New adenovirus genotypes are increasingly recognized through the use of phylogenetic analysis based on complete genomic sequences. Novel strains may arise from mutations or recombinations of two different adenovirus strains between the hexon gene and fiber genes.^3^,^4^ Recently, an emergent variant, HAdV-55,^5^,^6^ with a proposed recombination of hexon gene between HAdV-11 and HAdV-14 strains, has been described in association with multiple outbreaks of acute respiratory disease, mostly occurring in military camps.^7^–^10^ In 2005, a large outbreak of acute respiratory disease likely from HAdV-55 occurred in a military camp in Turkey.^8^ Another two HAdV-55 outbreaks occurred among military training camps in 2005 (226 patients in Singapore^9^) and in 2009 (108 patients in China^10^). Nucleotide sequence analysis showed the strains from the two outbreaks were highly similar to the QS-DLL strain, which was the first HAdV-55 in China isolated from an ARDS outbreak in Shaanxi in t 2006 that occurred in a senior high school.^7^ The most recent HAdV-55 outbreak occurred in February 2012 among patients with febrile respiratory tract infection admitted to PLA 252 hospital, Hebei Province, China (unpublished data). With rare precedent circulation of HAdV-11 and HAdV-14 in China, an increasing trend of adenovirus type 55 infections was observed among both civilian and military populations, probably due to the lack of immunity herd. This concern, in combination with its higher tendency in causing severe respiratory illness than other adenovirus, posed great threats to Chinese military that it has the potential to spread widely and cause severe epidemics. The status highlights the need for improved surveillance, with extensive molecular characterization, to identify the prevalence and the genetic variants of this emerging adenovirus. Thus far, HAdV surveillance in China is sparse, and HAdV-55 infections were chiefly identified during outbreak events. The current study was sought to acquire a better understanding of the prevalence and molecular evolution of HAdV-55 strains by performing an active surveillance for HAdV infections. This knowledge might assist with targeted population for disease prevention and geographic regions where type-specific vaccines should be administered if developed.

## Materials and methods

### Sample collection

From June 2009 to January 2012, we recruited hospitalized children with ARTI at Chongqing Children's hospital, Chongqing, China. ARTI was defined as acute onset of cough, rhinorrhea, and dyspnea, with fever ≥37·5°C. Nasopharyngeal aspirates (NPAs) were collected from the recruited children within 24 hours of admission and stored in the −80°C.

A standardized questionnaire was used to gather demographic and hospitalization data such as signs, symptoms, underlying medical conditions, and laboratory test results, radiographic findings, and disease outcome. Laboratory tests included sputum culture and an array of serological assays including IgG and IgM enzyme-linked immunosorbent assays (ELISAs) for previous infection with cytomegalovirus (CMV), Epstein–Barr virus (EBV), *Chlamydia pneumoniae* (CP), and *Mycoplasma pneumoniae* (MP).

### Detection of HAdV and other respiratory viruses

DNA and RNA were extracted from each specimen using the QIAamp® MinElute Virus Spin Kits (QIAGEN, Hilden, Germany). Molecular assays for HAdV were performed using pan primers as previously described.^11^ Specimens were screened for human bocavirus (HBoV), and influenza A virus by real-time RT-PCR^12^ as well as for respiratory syncytial virus (RSV), parainfluenza virus (PIV), human metapneumovirus (MPV), and coronavirus by nested PCR.^13^ Each PCR run included virus isolates DNA or RNA as positive control and water as negative control.

### Determination of HAdV genotypes

For HAdV generic PCR-positive samples, the highly variable regions of hexon gene and entire fiber gene were PCR amplified from viral DNA and then sequenced as previously described.^14^,^15^ Genomic sequences were assembled using CLC genomic workbench 5.1 (CLC bio, Aarhus, Denmark). Previously published GenBank sequences of HAdV, including HAdV-1, HAdV-3, HAdV-11, HAdV-14, HAdV-21, and HAdV-55, were used for comparison and determination of the HAdV genotypes. All comparison alignments were performed, and phylogenetic tree was constructed by neighbor-joining method with 1000 bootstrap replicates using CLC genomic workbench 5.1. Similarities between strains were calculated using BioEdit, version 7.13 (North Carolina State University, Raleigh, NC, USA; http://www.mbio.ncsu.eud/bioedit/bioedit.html).

### Molecular analysis of HAdV-55

For the determined HAdV-55, the whole genome sequences were obtained using an automated DNA sequencer (3730 DNA Sequencer; Applied Biosystems, Foster City, CA, USA). Hexon and fiber gene sequences of HAdV-55 available in the GenBank were collected for the establishment of phylogenetic tree for HAdV-55.

The rate of substitutions in HAdV-55 strains, their divergence over time, and the time to the most recent common ancestor (TMRCA) were estimated using a Bayesian Markov chain Monte Carlo (MCMC) approach as implemented in the Bayesian Evolutionary Sampling Tree package, version 1.7.2.^16^ The best-fit substitution model was chosen by performing a maximum likelihood analysis using the jModeltest package, version 0.1.1.^17^ Relaxed lognormal molecular clocks were employed and followed by allowing substitution rate variations among branches on the trees. The Bayesian MCMC chain lengths were 1 million generations with sampling every 1000 chain and discarding 10% of the chain as burn-in. Convergence of the chains was achieved by computational run over a sufficient time with inspection of MCMC samples using TRACER, version 1.5 (http://beast.bio.ed.ac.uk/). The resulting tree of each run was summarized using Tree Annotator, and the maximum clade credibility tree was visualized with FigTree software, version 1.3.1 (http://tree.bio.ed.ac.uk/software/figtree/). All GenBank accession numbers for the sequences obtained in this study, as well as those used for comparison are listed in Table 1.

**Table 1:** Reference sequences for the hexon/fiber genes and complete genomes from GenBank

Strain	Accession No. (Hexon/Fiber)	Strain	Accession No. (Complete genome)
South Dakota/6380/1997	FJ841899/FJ841907	HAdV-14	AY803294
Spain/273/1969	FJ841900/FJ841908	HAdV-11	AF532578
Taiwan/760/2002	FJ841905/FJ841913	HAdV-3	AY599834
Taiwan/2474/2001	FJ841906/FJ841914	HAdV-7	AY594255
Singapore/1218/2005	FJ607010/FJ603105	QS-DLL	FJ643676
Singapore/1223/2005	FJ607012/FJ603104	SGN1222	FJ597732
CQ-814	JX120175/JX120166	CQ-814	JX123027
CQ-1657	JX120175/JX120170	CQ-1657	JX123028
CQ-1674	JX120177/JX120167	CQ-2903	JX123029
CQ-1741	JX120172/JX120168	China/P14/2011	JX491639
CQ-1747	JX120173/JX120169	HAdV-16	AY601636
CQ-2903	JX120176/JX120171	HAdV-21	AY601633
HAdV-14p	FJ841903/FJ841911	HAdV-4	AY594253
		HAdV-26	EF153474
		HAdV-12	AC_000005
		HAdV-40	NC_001454
		HAdV-34	AY737797
		HAdV-35	AY128640
		HAdV-5	AY601635
		HAdV-52	DQ923122

## Results

Altogether 2234 hospitalized children were recruited in the study, their ages ranged from 1 month to 14 years (median 8 months) and 66·6% were male. Among all the tested children, 191 (8·55%) were infected with HAdV. Further genotyping revealed 92 (48·17%) infection with HAdV-7, 72 (37·70%) with HAdV-3, 6 (3·14%) with HAdV-55, 5 (2·62%) with HAdV-5, 4 (2·09%) with HAdV-1, 1 (0·52%) with HAdV-2, and 11(5·76%) with untyped HAdV (not sequenced due to running out of the samples).

### Genetic characterization of HAdV-55

**Figure 1:**
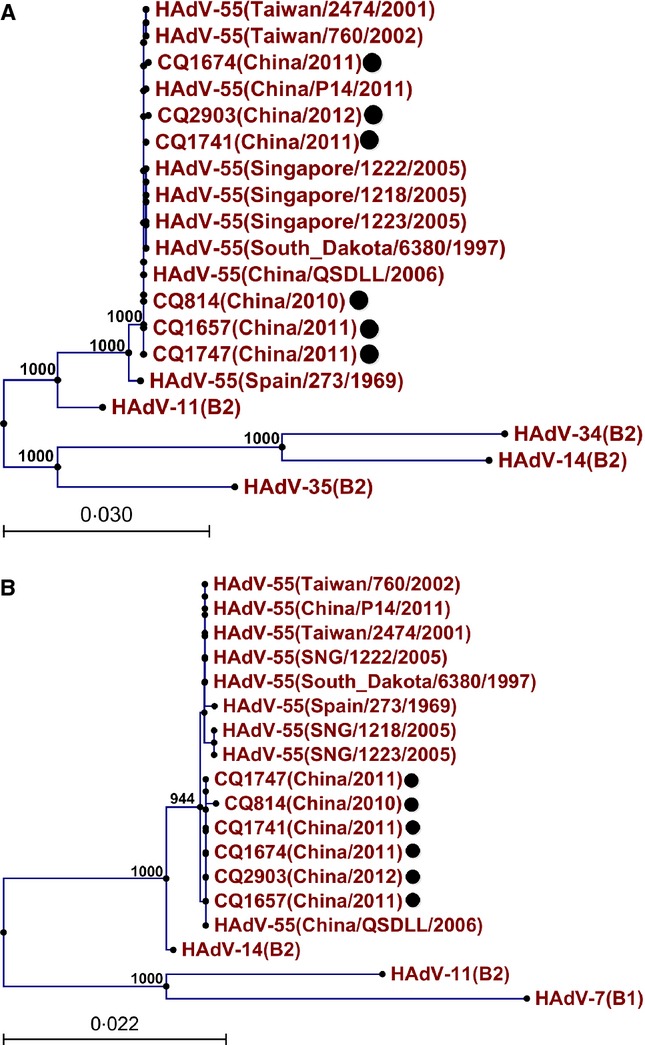
Phylogenetic analysis of human adenovirus based on partial hexon gene covering hypervariation regions 1–7 (nt 18353-20956, corresponding to the QS-DLL strain) and fiber gene (nt 30817-31752) was constructed using neighbor-joining method with 1000 bootstrap replicates. A, the hexon gene; B, the fiber gene. The strains in our study are labeled with the black solid circle.

The partial hexon gene covering hypervariation regions 1–7 (nt 18353-20956, corresponding to the QS-DLL strain) and fiber gene (nt 30817-31752) were successfully amplified from all the six samples, yielding 2604-bp and 954-bp amplicon, respectively. The sequence homologies of the six HAdV-55 strains were 99·8–100% for the hexon gene and 99·9–100% for the fiber gene, respectively. Phylogenetic analyses based on hexon gene and fiber gene revealed the HAdV strains in Chongqing clustered with HAdV-55 (Figure 1). Figure 1A shows that all these hexon genes obtained in this study clustered with HAdV-11 and exhibit the highest degree of nucleotide sequence identity with the strains in Singapore and USA, but different from the strains in Spain and Taiwan with the 1000 bootstraps supporting. Figure 1B demonstrates all these fiber strains from the mainland of China strains clustered with HAdV-14 strain as the expected appearance and form a separate branch, different from Taiwan and Singapore strains with the 897 bootstraps supporting. The phylogenetic trees based on hexon and fiber gene at amino acid level were also demonstrated in Figure S1, which were the same to those at nucleic acid level.

The homology analysis demonstrated all the determined nucleotide acid sequences and deduced amino acid sequences exhibited high homology to the QS-DLL strain for both hexon gene (99·9–100%) and fiber gene (99·8–100%). While lower homologies (90·7%) were revealed in hexon gene between the six strains and the HAdV-14 strain (GenBank Accession No.: AY803294) and in fiber gene (91·9%) between the six strains and the HAdV-11 strain (GenBank Accession No.: AF532578).

From the above results, three strains were sequenced for the whole genome sequences with the homology of 99·8%. The recombination analysis indicated that the HAdV-55 strain (CQ2903) shows a hexon recombination between HAdV-11 and HAdV-14 (Figure S2).

**Figure 2:**
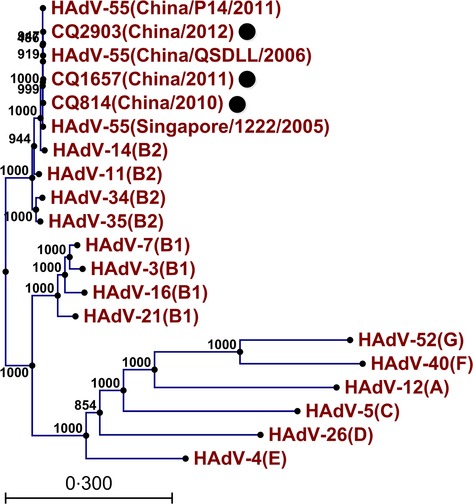
Phylogenetic tree of human adenovirus based on the whole genome sequence constructed by neighbor-joining method with 1000 bootstrap replicates. The strains in our study are labeled with the black solid circle.

**Figure 3:**
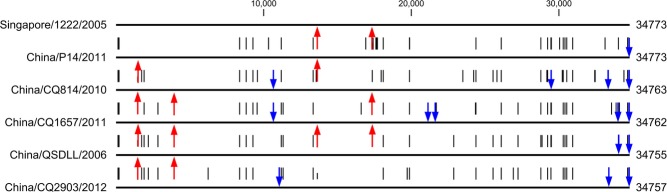
Variation sites based on the SGN1222 strain. The black line represents the mutation, the red up arrow represents base deletion, and the blue down arrow represents base insertion.

Phylogenetic tree for the whole genome sequences of HAdV-55 demonstrated that all previous HAdV-55 strains and our HAdV-55 strains clustered together with previously reported HAdV-14 strains (Figure 2). And the current viral strain, CQ-814 (Chongqing 2010 strain), was most closely related to P14 (Beijing 2011 strain), followed by CQ-1657 (Chongqing 2011 strain) and CQ-2903 (Chongqing 2012 strain). Surprisingly, the closest genetic relationship existed between CQ-2903 and QS-DLL (Shaanxi 2006 strain). The comparisons of variations for all the HAdV-55 whole genome sequences were shown in Figure 3. Most variations were missense mutations and silent mutations. The indels occurred frequently in the ends and 10 000–180 000 bases. The detailed variations among the whole genome sequences of HAdV-55 are listed in Table S1.

The overall evolutionary rates of hexon gene and fiber gene of HAdV-55 were estimated at 6·2 × 10^−5^ nucleotide substitutions per site per year (s/s/y) (95% HPD: 5·5 × 10^−6^–1·5 × 10^−4^ s/s/y) and 8·0 × 10^−5^ s/s/y (95% HPD: 6·6 × 10^−6^–1·8 × 10^−4^ s/s/y), respectively. The evolutionary rates were lower (1·7 × 10^−5^ s/s/y, 95% HPD: 2·2 × 10^−9^–3·5 × 10^−5^ s/s/y) when based on whole genome. The HAdV-55 strains from mainland China seems to have diverged from their TMRCA with other strains from Taiwan Province/Singapore/South Dakota around 15 years ago considering the hexon gene (Figure S3A), 10 years ago considering the fiber gene (Figure S3B), and 35 years ago considering the whole genome (Figure S3C), respectively.

### Clinical and laboratory characteristics of HAdV-55 infection

All the six patients with HAdV-55 infection were boys, and their ages ranged from 1 through 4 years old. All the patients occurred from August 2010 to January 2012. Three were diagnosed with severe pneumonia and/or toxic encephalopathy with their disease onset occurring in May 2011 (Table 2). The length of their hospitalization stay ranged from 4 to 12 days (median 7·5 days). Three patients had underlying medical conditions. In comparison with patients with HAdV-3 and HAdV-7 infection, patients with HAdV-55 infection presented with similar clinical manifestations (Table S2). All patients had fever of ≥38·5° with duration from 6 to 14 days, pharyngeal congestion, and swollen tonsils. The routine blood tests of all six patients were within normal ranges. Four patients had abnormal chest radiograph findings, which included increase in lung markings, interstitial change, or overinflation. The patients all recovered without sequela.

**Table 2:** The characteristics of the six hospitalized children infected with HAdV-55

Patient	Age	Sex	Sample	Onset date	Clinical diagnosis	Underlying conditions	Coinfection
1	11 y 1 mo	boy	CQ-814	August 15, 2010	URTI	No	MPV
2	1 y 4 mo	boy	CQ-1657	April 22, 2011	Interstitial pneumonia	Wheezing eczema	MPV, PIV-1, *Haemophilus influenza*
3	1 y 4 mo	boy	CQ-1674	May 1, 2011	Protracted pneumonia Toxic encephalopathy Bronchial asthma	No	HBoV, *Staphyloccocus aureus*
4	1 y 2 mo	boy	CQ-1741	May 9, 2011	Severe pneumonia Respiratory failure	Wheezing	HBoV, PIV-1, CMV-IgM (+), *Klebsiella pneumonia*
5	4 y 0 mo	boy	CQ-1747	May 17, 2011	Severe pneumonia Toxic encephalopathy	No	PIV-1, CP-IgM (+)
6	3 y 4 mo	boy	CQ-2903	January 8, 2012	Bronchitis	Urticarial	No

y, year; mo, month; CQ, Chongqing; URTI, upper respiratory tract infection; CP, chlamydia pneumonia; MPV, metapneumovirus; PIV, parainfluenza virus; HBoV, human bocavirus; CMV, cytomegalovirus.

### Microbiological investigation

Coinfections were frequent among the 6 hospitalized boys infected with HAdV-55. Three were coinfected with one other virus (one with MPV, one with HBoV, and one with PIV-1). Two were coinfected with two other viruses (both with HBoV and PIV-1). Three were coinfected with bacteria (*Haemophilus influenz*ae, *Staphyloccocus aureus,* and *Klebsiella pneumoniae*, respectively). Patient-4 also had evidence of recent CMV infection (IgM assay). Patient-5 was similarly positive for recent CP infection (IGM assay).

## Discussion

In the United States, HAdV strains of low type number are highly prevalent among children with HAdV-3, and HAdV-7, a frequent cause of outbreaks.^18^,^19^ Recently, novel recombination strains, including HAdV-55 and HAdV-14p1, have been evolved from a recombination of HAdV-11 and HAdV-14 ancestral strains.^20^

In Asia, the prevalence of HAdV has ranged from 0·8% to 11·30% among patients with ARTI.^21^,^22^ In the past, HAdV-3 and HAdV-7 strains have been the most prevalent HAdV types.^1^,^23^ However, recent outbreaks have also documented high prevalence of HAdV-5^24^–^27^ and HAdV-55.^7^–^10^ Our study provides further evidence that HAdV-55 is likely routinely circulating among Chinese children. In this hospital surveillance study, we documented six hospitalized patients with HAdV-55 infection. Our whole genome sequence data confirm the relatedness of our HAdV-55 isolates with HAdV-14, which classically has caused outbreaks (sometimes with severe disease) in various regions of North America,^18^ Europe,^28^, and Asia.^10^ Our HAdV-55-infected patients had severe or protracted pneumonia as well as toxic encephalopathy.^20^–^26^,^28^,^27^ However, whether HAdV-55 was responsible for the clinical manifestation cannot be determined, because 5 of the 6 patients with human HAdV-55 infections had also been coinfected with other pathogens.

Recombination is a well-known feature in HAdV genetics and an important force driving the evolution of HAdV.^1^,^2^ Our study further supported the hypothesis that HAdV-55 is a recombinant between HAdV-11 and HAdV-14 strains and more HAdV-14-like than HAdV-11-like.^5^ Meanwhile, Based on the phylogenetic trees for hexon gene, the Singapore strains are more closely related to the mainland China HAdV-55 strains than with Taiwan strains while the opposite to the fiber gene. These results suggested a possible recombination event occurred at a position between hexon gene and fiber gene, between mainland China strain and Taiwan strain, which resulted in the formation of the Singapore strain. All the strains in mainland of China seem to be different from other regional HAdV-55 strains. These characteristics might reflect either spatial or temporal dynamics of HAdV-55.

For phylogenetic tree of the whole genome, the closest genetic relationship was found between the 2012 Chongqing strain (CQ-2903) and the 2006 Shaanxi strain (QS-DLL). The reason of this close relationship needs to be illustrated. A possible explanation is that all these HAdV-55 strains are derived from different lineages of a common ancestor. Data from the phylogenetic tree and molecular clock analysis suggest HAdV-55 evolved with a certain evolution rate and geographic subdivision occurred during the last 35 years, as well as Chongqing HAdV-55 strains were accumulating mutations at a certain speed from 2010 to 2012. Further molecular investigation based on HAdV-55 of wider origin might facilitate to understand its dissemination and transmission in China.

Given the possible association of severe disease with HAdV-55, the possible emergence of other novel adenovirus recombinants, and our sparse understanding of HAdV epidemiology in China, we posit that more aggressive population-based surveillance for HAdV strains should be conducted such that, if warranted in the future, type-specific diagnostics,^30^ new antiviral therapeutics,^31^ and possibly adenovirus vaccines^19^ might be developed, studied, and employed among China's populations at highest risk of severe HAdV disease.

## Supporting Information

**Figure S1.** Phylogenetic analysis of human adenovirus based on partial hexon gene covering hypervariation regions 1–7 (nt 18353-20956, corresponding to the QS-DLL strain) and fiber gene (nt 30817-31752) at amino acid level was constructed using neighbor-joining method with 1000 bootstrap replicates.

**Figure S2.** The recombination analysis for HAdV-55 (CQ2903) based on the whole genome sequence by RDP 3.5.1.

**Figure S3.** Bayesian timescale phylogeny of HAdV-55 based on the hexon gene (A), fiber gene (B), and whole genome (C).

**Table S1.** The variations among the whole genome sequences of HAdV-55.

**Table S2.** Clinical manifestations and laboratory findings for the hospitalized children infected with HAdV.
