# Supplementary figures and images for: Epidemiology of human adenovirus and molecular characterization of human adenovirus 55 in China, 2009–2012
Source: Influenza Other Respir Viruses. 2014 Jan 28;8(3):302–8. doi: 10.1111/irv.12232 (PMC4181478; doi:10.1111/irv.12232)

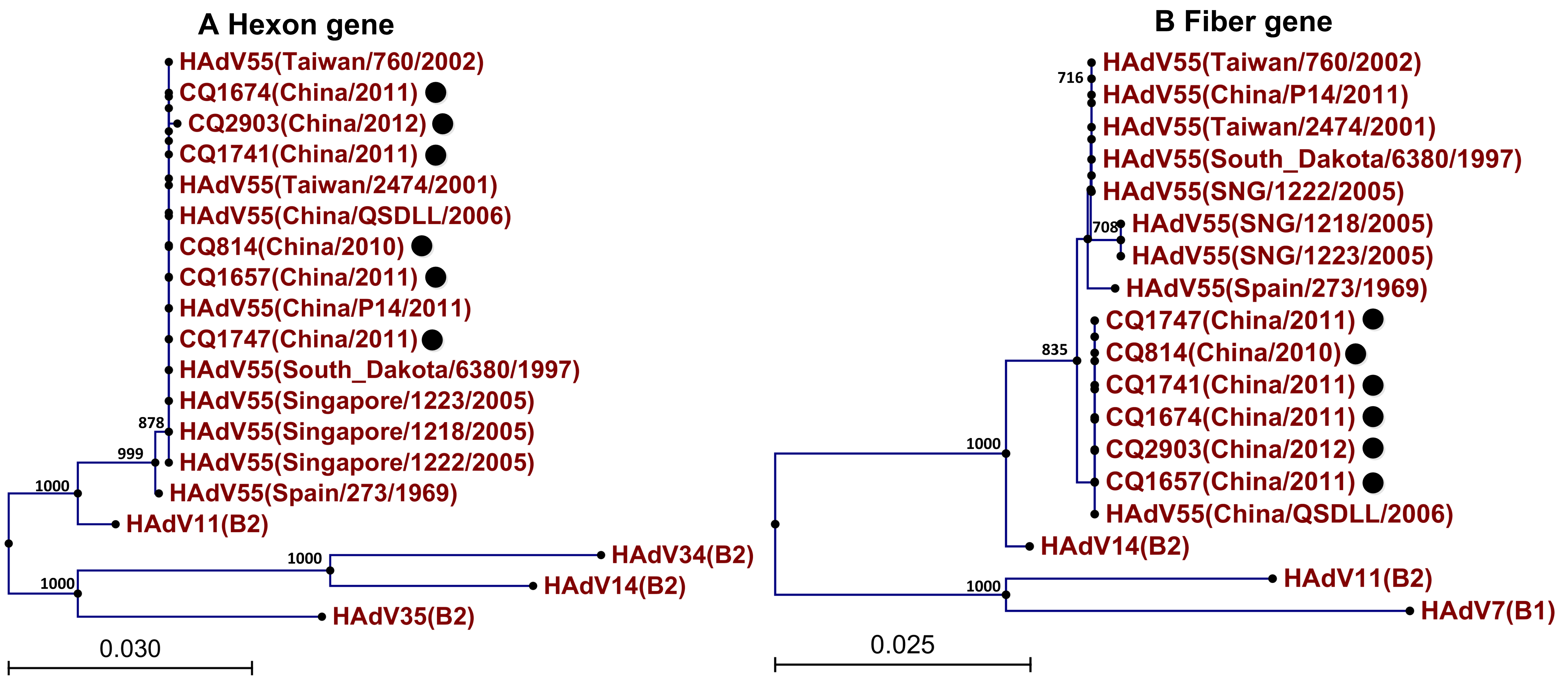

Supplement: Supplementary file 1 — Figure S1. Phylogenetic analysis of human adenovirus based on partial hexon gene covering hypervariation regions 1–7 (nt 18353-20956, corresponding to the QS-DLL strain) and fiber gene (nt 30817-31752) at amino acid level was constructed using neighbor-joining method with 1000 bootstrap replicates. [file irv0008-0302-SD1.tif]

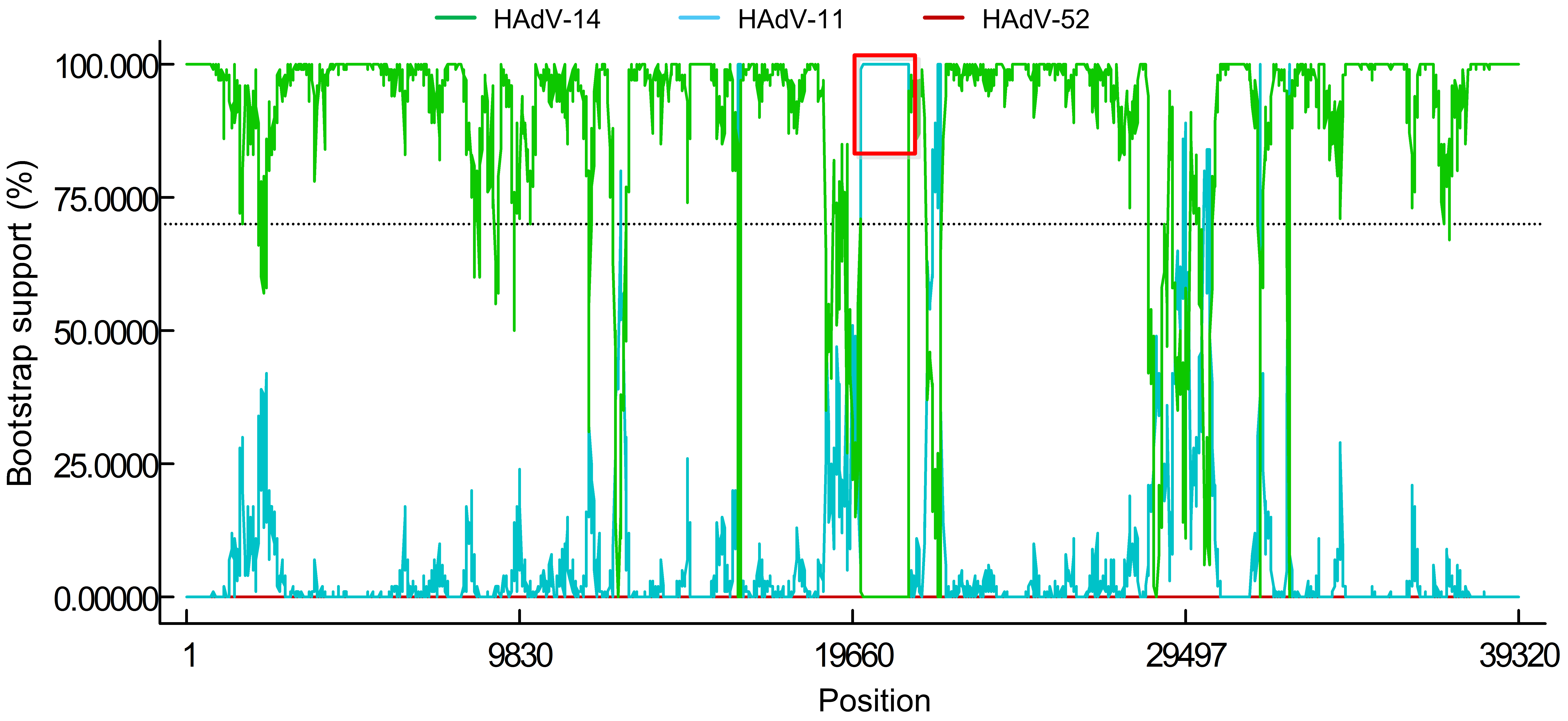

Supplement: Supplementary file 2 — Figure S2. The recombination analysis for HAdV-55 (CQ2903) based on the whole genome sequence by RDP 3.5.1. [file irv0008-0302-SD2.tif]

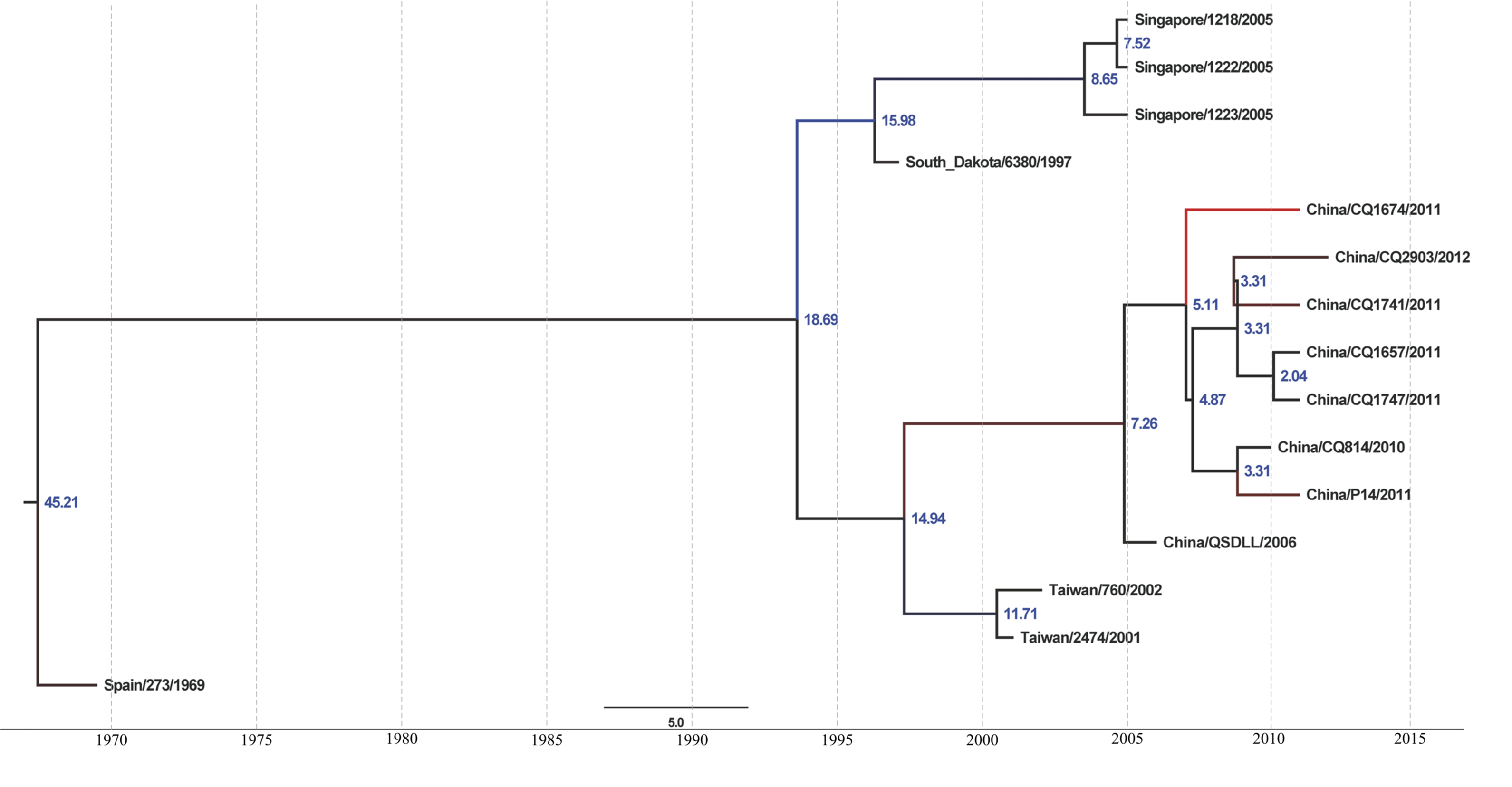

Supplement: Supplementary file 3 — Figure S3. Bayesian timescale phylogeny of HAdV-55 based on the hexon gene (A), fiber gene (B), and whole genome (C). [file irv0008-0302-SD3.tif]

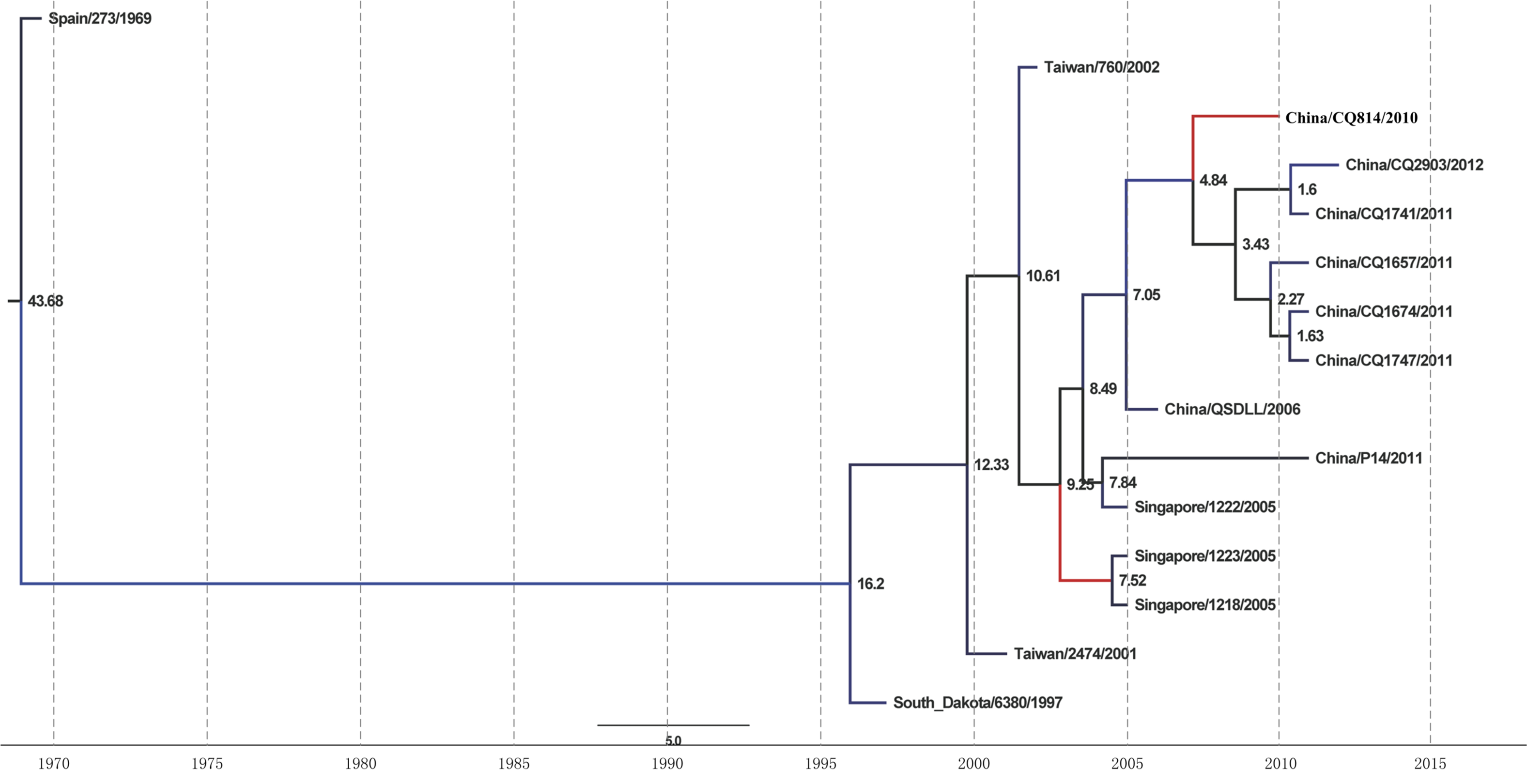

Supplement: Supplementary file 4 — Figure S3. Bayesian timescale phylogeny of HAdV-55 based on the hexon gene (A), fiber gene (B), and whole genome (C). [file irv0008-0302-SD4.tif]

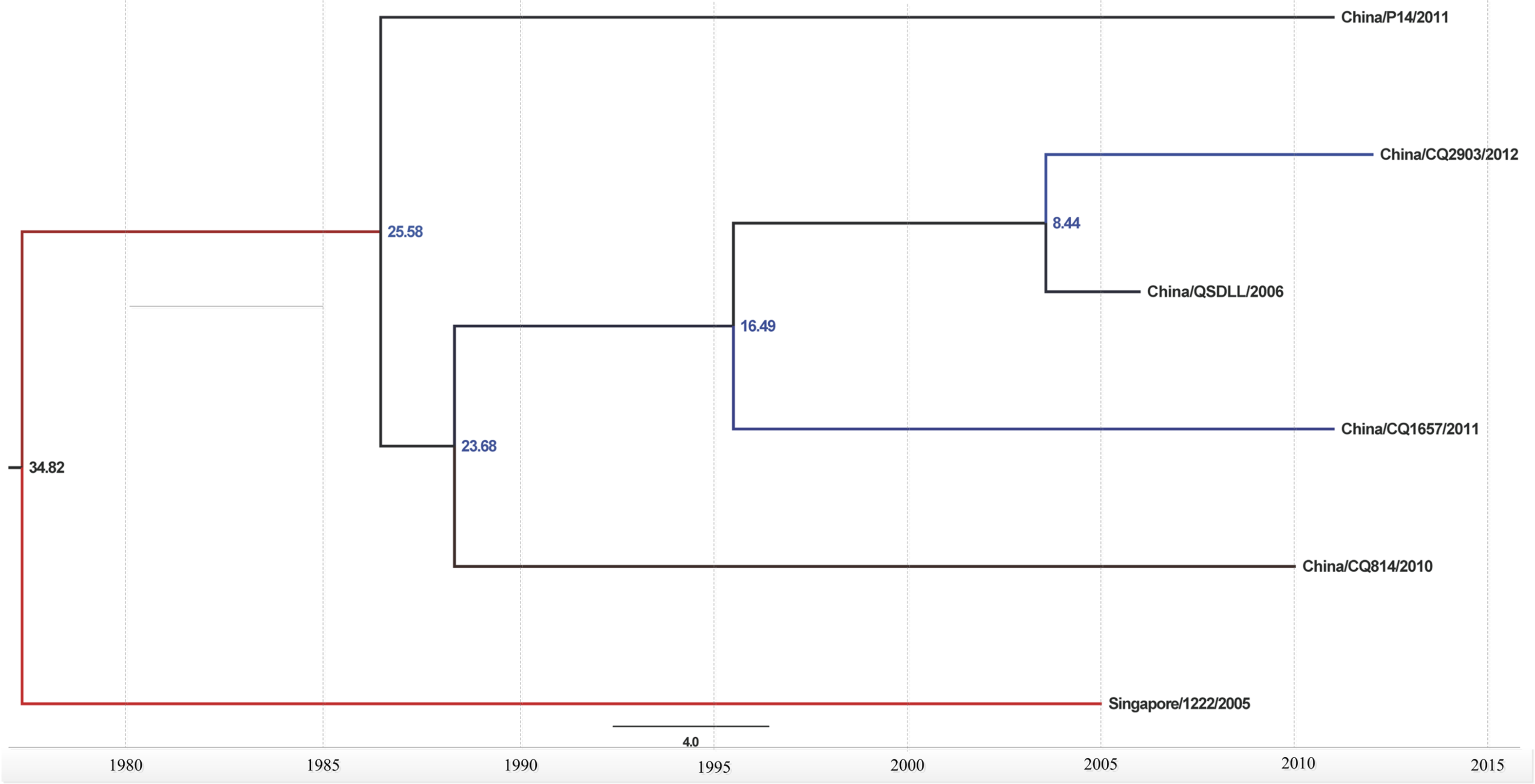

Supplement: Supplementary file 5 — Figure S3. Bayesian timescale phylogeny of HAdV-55 based on the hexon gene (A), fiber gene (B), and whole genome (C). [file irv0008-0302-SD5.tif]
