# Supplementary material for: Epidemiology of human adenovirus and molecular characterization of human adenovirus 55 in China, 2009–2012
Source: Influenza Other Respir Viruses. 2014 Jan 28;8(3):302–8. doi: 10.1111/irv.12232 (PMC4181478; doi:10.1111/irv.12232)
Supplement: Supplementary file 6 — Table S1. The variations among the whole genome sequences of HAdV-55. [file irv0008-0302-SD6.docx]

Supplemental Table S1. The variations among the whole genome sequences of HAdV-55

| **No.** | **Position  based on  SGN1222** | **Gene** | **QSDLL** | **CQ814** | **CQ1657** | **CQ2903** | **P14** | **SGN1222** |
| --- | --- | --- | --- | --- | --- | --- | --- | --- |
| 1 | 134 | ITR | C | C | C | C | C | T |
| 2 | 202 | Non-coding | C | C | C | C | C | G |
| 3 | 588 | E1A | - | - | - | - | - | A |
| 4 | 1466 | Non-coding | - | - | - | - | CCATATCCGTGTT | CCATATCCGTGTT |
| 5 | 1735 | E1B | A | T | T | A | T | T |
| 6 | 1899 | E1B | T | C | T | T | C | C |
| 7 | 2184 | E1B | T | C | C | T | C | C |
| 8 | 2837 | E1B | A | G | A | A | G | G |
| 9 | 3927 | Non-coding | - | AAAAAA | - | - | AAAAAA | AAAAAA |
| 10 | 6236c | E2B | G | G | G | A | G | G |
| 11 | 8371c | E2B | C | C | C | C | C | T |
| 12 | 8372c | E2B | A | A | A | A | G | A |
| 13 | 8815c | E2B | C | C | C | C | C | T |
| 14 | 9273c | E2B | C | C | C | C | C | G |
| 15 | 9556c | E2B | G | G | A | G | G | G |
| 16 | 9577c | E2B | G | A | G | G | G | G |
| 17 | 10329 | L1 | G | G | G | G | A | G |
| 18 | 10663 | L1 | - | --T | TTT | - | - | - |
| 19 | 11065 | L1 | - | - | A | - | - | - |
| 20 | 11187 | L1 | C | C | C | C | C | G |
| 21 | 11314 | L1 | G | A | G | G | A | A |
| 22 | 13348 | L1 | G | G | G | G | G | A |
| 23 | 13552 | L1 | G | A | G | G | G | G |
| 24 | 13629 | Non-coding | - | - | A | - | - | A |
| 25 | 16601 | L2 | G | G | A | G | G | G |
| 26 | 16917 | C | C | C | C | C | T | C |
| 27 | 17331 | Non-coding | AA | AA | -- | AA | -- | AA |
| 28 | 17350 | Non-coding | -- | AA | A- | A- | A- | A- |
| 29 | 17401 | Non-coding | G | G | G | G | A | G |
| 30 | 17410 | Non-coding | G | G | G | G | G | G |
| 31 | 17600 | Non-coding | C | C | C | C | G | C |
| 32 | 17606 | L3 | G | G | G | G | A | G |
| 33 | 17625 | L3 | G | G | G | G | A | G |
| 34 | 17644 | L3 | G | G | G | G | T | G |
| 35 | 17670 | L3 | T | T | T | T | G | T |
| 36 | 17703 | L3 | G | G | G | G | C | G |
| 37 | 17954 | L3 | G | A | G | G | G | G |
| 38 | 18094 | L3 | T | T | T | T | T | C |
| 39 | 19722 | L3 | C | C | T | C | C | C |
| 40 | 19877 | L3 | C | C | C | C | C | T |
| 41 | 19888 | L3 | T | T | T | T | C | T |
| 42 | 21128 | L3 | - | - | AGC | - | - | - |
| 43 | 21683 | L3 | C | C | G | C | C | C |
| 44 | 22541c | E2A | A | A | A | G | A | A |
| 45 | 22868c | E2A | A | C | C | A | C | C |
| 46 | 23484 | L4 | T | A | T | T | T | T |
| 47 | 24216 | L4 | A | T | A | A | A | A |
| 48 | 24340 | L4 | C | C | T | C | C | C |
| 49 | 24370 | L4 | T | T | T | T | T | A |
| 50 | 25531 | L4 | A | A | G | A | G | G |
| 51 | 25794 | L4 | C | T | C | C | C | C |
| 52 | 26082 | L4 | C | C | C | C | C | T |
| 53 | 26902 | L4 | C | C | C | T | C | C |
| 54 | 27220 | L4 | T | C | T | T | C | C |
| 55 | 28767 | E3 | C | C | C | C | C | T |
| 56 | 28849 | E3 | G | A | A | A | A | A |
| 57 | 29176 | E3 | A | G | A | A | A | A |
| 58 | 29226 | E3 | A | A | A | A | A | G |
| 59 | 29451 | E3 | T | T | T | T | T | C |
| 60 | 29483 | Non-coding | TT- | T-- | TTT | --- | TT- | --- |
| 61 | 30049 | E3 | G | G | G | G | T | G |
| 62 | 30216 | E3 | C | T | C | C | C | C |
| 63 | 30279 | E3 | G | G | G | G | G | A |
| 64 | 30398 | E3 | A | G | A | A | A | G |
| 65 | 30521 | E3 | G | G | G | G | G | A |
| 66 | 30921 | L5 | G | G | G | G | G | C |
| 67 | 31484 | L5 | T | C | T | T | T | T |
| 68 | 32413c | E4 | C | T | C | C | C | C |
| 69 | 32443c | E4 | T | C | T | T | T | T |
| 70 | 33124c | E4 | C | C | C | C | T | C |
| 71 | 33319c | E4 | - | T | - | - | - | - |
| 72 | 33388 | E4 | - | - | - | A | - | - |
| 73 | 33550c | E4 | G | G | A | G | G | G |
| 74 | 33858c | E4 | G | G | A | G | G | G |
| 75 | 34012c | E4 | A | - | A | - | A | - |
| 76 | 34084c | E3 | G | G | A | G | G | G |
| 77 | 34389c | E3 | C | G | C | C | C | C |
| 78 | 34636c | ITR | G | G | G | G | G | A |
| 79 | 34715c | ITR | T | T | T | T | T | - |
