# Supplementary material for: Epidemiology of human adenovirus and molecular characterization of human adenovirus 55 in China, 2009–2012
Source: Influenza Other Respir Viruses. 2014 Jan 28;8(3):302–8. doi: 10.1111/irv.12232 (PMC4181478; doi:10.1111/irv.12232)
Supplement: Supplementary file 7 — Table S2. Clinical manifestations and laboratory findings for the hospitalized children infected with HAdV. [file irv0008-0302-SD7.docx]

Supplemental Table S2 Clinical manifestations and laboratory findings for the hospitalized children infected with HAdV

| Characteristic | HAdV-55  (n=6) | HAdV-3  (n=72) | HAdV-7  (n=92) | *P* value | |
| --- | --- | --- | --- | --- | --- |
|  |  |  |  | 55 vs. 3 | 55 vs. 7 |
| Age (month, median) | 28(14-133) | 16(1-121) | 14(1-127) | 0.165 | 0.073 |
| Sex (male) number(%) | 6(100) | 44(61.1) | 65(70.7) | 0.083 | 0.183 |
| Hospitalized duration(day, median) | 7.5(4-12) | 5.5(1-18) | 7.5(1-72) | 0.705 | 0.211 |
| Symptom and signs |  | Number (%) |  |  |  |
| Cough | 5(83.3) | 64(88.9) | 88(95.7) | 0.533 | 0.276 |
| Nasal discharge | 1(16.7) | 14(19.4) | 11(12.0) | 1.000 | 0.553 |
| Expectoration | 5(83.3) | 42(58.3) | 77(83.7) | 0.393 | 1.000 |
| Dyspnea | 2(33.3) | 8(11.1) | 28(30.4) | 1.000 | 0.178 |
| Diarrhea | 2(33.3) | 13(18.1) | 31(33.7) | 0.325 | 1.000 |
| Rhonchi | 3(50.0) | 27(37.5) | 45(48.9) | 0.671 | 1.000 |
| Moist rales | 4(66.7) | 47(65.3 | 73(79.4) | 1.000 | 0.606 |
| Laboratory test |  | Mean±SD |  |  |  |
| White blood cell (×10^9^/L) | 3.9±2.4 | 4.6±2.3 | 4.9±2.5 | 0.445 | 0.354 |
| Red blood cell (×10^9^/L) | 4.3±0.5 | 4.6±2.6 | 4.3±0.5 | 0.833 | 0.922 |
| Neutrophils (%) | 62.8±14.4 | 52.7±20.5 | 55.6±18.0 | 0.242 | 0.338 |
| Lymphocyte (%) | 33.3±13.8 | 42.5±19.8 | 39.9±16.7 | 0.270 | 0.353 |
| HGB (g/L) | 111±19 | 111±13 | 111±12 | 0.966 | 0.951 |
| PLT (×10^9^/L) | 270±103 | 336±144 | 268±150 | 0.273 | 0.979 |
| Other pathogens |  | Number (%) |  |  |  |
| Influenza virus | 0(0) | 12(16.7) | 6(6.5) | 0.583 | 1.000 |
| Respiratory syncytial virus | 0(0) | 11(15.3) | 12(13.0) | 0.586 | 1.000 |
| Parainfluenza virus | 3(50.0) | 10(13.9) | 16(17.4) | 0.055 | 0.085 |
| Coronavirus | 0(0) | 2(2.8) | 2(2.2) | 1.000 | 1.000 |
| Metapneumovirus | 2(33.3) | 2(2.9) | 1(1.1) | 0.030 | 0.010 |
| Human bocavirus | 2(33.3) | 14(19.4) | 11(12.0) | 0.0597 | 0.179 |
| CMV-IgM | 1(16.7) | 6(8.3) | 9(9.8) | 0.442 | 0.485 |
| EBV-IgM | 0 | 5(6.9) | 5(5.4) | 1.000 | 1.000 |
| CP-IgM | 1(16.7) | 5(6.9) | 4(4.4) | 0.392 | 0.276 |
| MP-Ab | 0 | 6(8.3) | 3(3.3) | 1.000 | 1.000 |
| *Staphyloccocus aureus* | 1(16.7) | 6(11.1)^a^ | 13(19.7)^b^ | 0.541 | 1.000 |
| *Haemophilus influenz*a | 1(16.7) | 4(7.4)^a^ | 3(4.6)^b^ | 0.421 | 0.299 |
| *Klebsiella pneumonia* | 1(16.7) | 3(5.6)^a^ | 1(1.5)^b^ | 1.000 | 1.000 |
| Outcome |  | Number (%) |  |  |  |
| Severe pneumonia | 2(33.3) | 14(19.4) | 42(45.7) | 0.597 | 0.688 |

a: Among the HAdV-3 positive patients, 18 were not cultured for the bacteria.

b: Among the HAdV-7 positive patients, 26 were not cultured for the bacteria.
